# Supplementary material for: Contraction reserve in high resolution manometry is correlated with lower esophageal acid exposure time in patients with normal esophageal motility: A retrospective observational study
Source: PLoS One. 2023 Nov 2;18(11):e0291010. doi: 10.1371/journal.pone.0291010 (PMC10621955; doi:10.1371/journal.pone.0291010)
Supplement: Attachment — Submitted filename: Response to Reviewers.docx [file pone.0291010.s003.docx]

Dear reviewers:

Thank you very much for your valuable comments and suggestions, which have provided great help in improving our manuscript. We have carefully revised the manuscript. Our responses to the reviewers’ comments are shown below:

Reviewer 1#

**1) What is the value of contraction reserve in normal HRM? Is the esophagus supposed to contract even more than he normally contracts? If I have normal motility is it not an assumption that I have contraction reserve?**

Response: It is reported that supine contraction reserve is existed in 70-80% of healthy volunteers while exist only in 46-50% of patients with normal HRM. Hence, it is supposed that the absence of contraction reserve may indicate potential damage to esophageal contractility. In the present study, we found that contraction reserve can independently predict abnormal AET. We discussed this question in the discussion part in the revised manuscript (page11, line 202-208).

**2) Why would reserve affect AET in normal peristalsis if esophageal clearance is theoretically normal?**

Response: Impaired esophageal clearance is not the only reason for abnormal AET. Transient lower esophageal sphincter (LES) relaxation, lower mean basal LES pressure, decreased postreflux swallow-induced peristaltic wave index, etc., are all risks factors for pathological acid exposure. The mechanism by which contraction reserve affects AET is not clear at present. We suppose that in some patients, the absent of contraction reserve may indicate potential abnormality of esophageal motility. However, in other patients, it may not have specific significance because 20-30% of healthy volunteers do not have contraction reserve.

**3) Contraction reserve could be useful in patients with IEM to show that despite abnormal motility with standard stimulation, there is still working muscle with overload. The authors did not saw a correlation of this with GERD. Then what is the use of this parameter?? The authors are deyifing this parameter.**

Response: We designed this study because we doubted the value of contraction reserve in predicting abnormal acid reflux. We finally found a correlation between contraction reserve and abnormal AET in patients with normal esophageal motility, but not IEM. The mechanism by which contraction reserve affects AET is not clear.

**4) Language needs some review in terms of scientific soundable. E.g. "Ineffective esophageal motility (IEM) is most often encountered in patients referred for high-resolution manometry" --> that is obvious. If the diagnosis is made by HRM how can patients be diagnosed with IEM otherwise?**

Response: We have carefully revised the language of the entire manuscript. This sentence has been revised as “Ineffective esophageal motility (IEM) is the most common esophageal dysmotility diagnosed by high-resolution manometry.”

**5) Why esophagitis LA C was excluded?**

Response: At the design stage, we excluded LA C esophagitis to avoid the possibility that severe esophagitis induced esophageal dysmotility, which might influence the study results. The only LA C esophagitis is normal HRM with supine contraction reserve absent and uptight contraction reserve present. The total AET and supine AET were 1.1% and 1.2% respectively. When this case is included in the analysis, the result is not changed. Therefore, we did not revise the study protocol to include this case.

**Reviewer 2#**

1) In the discussion section, page 3, lines 232 on, please briefly comment a recent review paper (Riva CG, et al, J Neurogastro Motility 2020;26(3):335-343) focusing on the drawbacks of conducting HRM studies in the supine position only.

Response: We have revised the manuscript accordingly. Discrepancies in HRM diagnosis between upright and supine position are not rare. Final HRM diagnosis is recommended to be made based on both supine and upright position which can decrease clinically irrelevant diagnosis. The revised text can be found in page 12, line 229-232.

2) Please check the following items: page 8, line 140, "...patients had...", line 147, delete "...tendency toward..."; page 9, line 162, delete "tendency toward", line 168, delete "...trended strongly"; page 13, line 239, "...patients had...; page 14, line 270, "...the present study does not support....

Response: We have revised the manuscript accordingly.

Thank you very much for your attention for this manuscrip. We look forward to hearing from you soon.

Best regards,

Corresponding author:

Yi ZH, MD

Gastroenterology department of West China Hospital, Sichuan University

Chengdu, Sichuan province, P.R.China

Email: yizhihuiHX@163.com
